# Laboratory evaluation of eleven rapid diagnostic tests for serological diagnosis of Chagas disease in Colombia

**DOI:** 10.1371/journal.pntd.0011547

**Published:** 2023-08-22

**Authors:** Andrea Marchiol, Astrid Carolina Florez Sanchez, Andrés Caicedo, Maryi Segura, Jessica Bautista, Martha Stella Ayala Sotelo, Rafael Herazo, Colin Forsyth, Laura C. Bohorquez

**Affiliations:** 1 Drugs for Neglected Diseases initiative-Latin America, Rio de Janeiro, Brazil; 2 Dirección de Redes de Laboratorio, Insitituto Nacional de Salud, Bogotá, Colombia; 3 Departamento de Parasitología, Instituto Nacional de Salud, Bogotá, Colombia; 4 Foundation for Innovative New Diagnostics, Geneva, Switzerland; University of North Carolina at Chapel Hill School of Medicine, UNITED STATES

## Abstract

**Background:**

Chagas disease is a public health challenge in Colombia, where only an estimated 1.2% of people at risk have accessed diagnosis, while less than 0.5% of affected people have obtained treatment. The development of simplified diagnostic algorithms would enable progress in access to diagnosis; however, the current diagnostic algorithm relies on at least two laboratory-based tests that require qualified personnel, processing equipment, and infrastructure, which are still generally lacking at the primary care level. Rapid diagnostic tests (RDTs) for Chagas disease could simplify diagnosis, but their performance in the epidemiological context of Colombia is not well known.

**Methodology:**

A retrospective analytical observational study of RDTs was performed to estimate the operational characteristics of 11 commercially available RDTs designed for in vitro detection of anti-*T. cruzi* IgG antibodies. The study was performed under controlled laboratory conditions using human serum samples.

**Principal findings:**

Eleven RDTs were assessed, ten using 585 serum samples and one using 551 serum samples. Employing the current national diagnostic algorithm as a reference standard for serological diagnosis of chronic infection, the sensitivity of the assessed RDTs ranged from 75.5% to 99.0% (95% CI 70.5–100), while specificity ranged from 70.9% to 100% (95% CI 65.3–100). Most tests (7/11, 63.6%) had sensitivity above 90%, and almost all (10/11, 90.9%) had specificity above 90%. Five RDTs had both sensitivity and specificity above 90%.

**Conclusions/Significance:**

The evaluation of these 11 commercially available RDTs under controlled laboratory conditions is a first step in the assessment of the diagnostic performance of RDTs in Colombia. As a next step, field studies will be conducted on available RDTs with sensitivity and specificity greater than 90% in this study, to evaluate performance in real world conditions, with the final goal to allow simplified diagnostic algorithms.

## Author summary

Chagas disease is an infectious parasitic disease affecting more than 400,000 people in Colombia. The Pan American Health Organization (PAHO) has estimated that 70% of infected people do not present clinical symptoms. To determine if a person has the infection, blood tests must be performed in laboratories that are generally not located near primary health care centers. Although diagnostic coverage has improved recently, the way Chagas disease is diagnosed needs to be further simplified in order to test more people for the infection and provide early treatment. For this, rapid diagnostic tests (RDTs) are needed. RDTs are small devices that, using a drop of blood taken from a finger, can determine in a few minutes whether a person is likely to have *T*. *cruzi* infection, the parasite responsible of Chagas disease. These tests can be quickly performed while a person receives care, even outside a hospital or medical center, because they are easy to use and do not need specialized training to be done. There are several commercially available RDTs for *T*. *cruzi* detection, but more information is needed about their performance in diagnosing the disease in the Colombian population. For this reason, 11 RDTs, commercially available in the Latin American region, have been assessed under controlled laboratory conditions, to determine their ability to diagnose *T*. *cruzi* infection in the Colombian population. This study found seven RDTs which are more than 90% effective in diagnosing Chagas disease. Now, those RDTs with the best diagnostic performance need to be evaluated in the real world in field studies, outside controlled laboratory conditions. If the field performance of these tests proves to be optimal (i.e. comparable to the current laboratory-based diagnostic methods), RDTs could be incorporated into the Colombian diagnostic protocol, thus allowing decentralization of diagnosis and helping improve access and health care coverage for people at risk of suffering from Chagas disease.

## Introduction

Chagas disease, one of 20 conditions classified as neglected tropical diseases (NTDs) by the World Health Organization (WHO) [1], affects more than 6 million people worldwide, mainly in endemic areas of Latin America, and is largely underdiagnosed. According to estimates, less than 10% of those currently infected know their infection status [2], which represents a significant barrier to receive timely comprehensive care. Less than 1% of the estimated population with the infection receives etiological treatment. Awareness is a key issue, as most patients are asymptomatic and unaware of their infection, and healthcare providers are often unfamiliar with the disease and its risk factors. Facilitating testing at point of care, in addition to amplifying access to information about the disease, could stimulate increased testing and access to treatment. Timely treatment sustainably eliminates parasitaemia, which can prevent disease progression and complications in some cases or prevent congenital transmission and cure the disease in others [3–7].

In Colombia, Cucunubá and colleagues (2017) found that only 1.2% of the population considered at risk by the WHO [8] had been screened for Chagas disease, and only 0.4% of an estimated 438,000 infected persons had access to etiological treatment [9]. About a third of individuals who received a positive test result were not able to obtain a complementary test to confirm their diagnosis.

In 2015, the Colombian Ministry for Health and Social Protection, the Colombian National Institute of Health (INS), and the Drugs for Neglected Diseases initiative (DNDi) started an inter-institutional collaboration to improve the diagnosis and treatment of Chagas disease. The process began with a barrier identification workshop that gathered important inputs for the development and validation of a new comprehensive care roadmap (CCR) specific to Chagas disease, including proposed solutions for eliminating the identified barriers [10]. Key bottlenecks identified included delayed or missed diagnostic confirmation with a second serological test [8]. At this time, the diagnostic algorithm involved one of various enzyme-linked immunosorbent assays (ELISAs) as an initial test, followed by an indirect immunoflourescence assay (IFA), or alternatively an indirect hemagglutination assay (IHA), as a complementary test. The IFA test was often not available near where patients lived and received healthcare, posing an important barrier to diagnosis.

In response to this finding, a new algorithm based on two different ELISAs and an IFA as a tiebreaker in case of discordance, was developed and included in the CCR. After 30 months of implementation, in the five municipalities where the care roadmap was validated, 5,654 people were tested, and 649 people with *T*. *cruzi* infection were identified, resulting in a 5.6-fold increase in the number of patients diagnosed, a 7-fold increase in the probability of detecting infected patients and a reduction from 258 to 19 days in the waiting time for diagnostic confirmation, compared to a baseline assessment before implementation [11]. The CCR including the new diagnostic algorithm has since been expanded to several other endemic municipalities in Colombia.

The chronic phase of Chagas disease is diagnosed by detecting circulating IgG antibodies for *T*. *cruzi*. There are several laboratory-based diagnostic tests based on different immunological principles, such as IFA, IHA, ELISA, and chemiluminescence assay (CLIA), all of which are used in clinical practice.

The Pan American Health Organization (PAHO) recommends at least two positive serological laboratory-based test results to confirm diagnosis of chronic *T*. *cruzi* infection [12]. The laboratories performing such tests need to have qualified personnel, specific processing equipment, and infrastructure that are generally not available at the primary care level in endemic areas. Currently, there are several commercially available rapid diagnostic tests (RDTs) for chronic *T*. *cruzi* infection which detect specific antibodies using whole blood, plasma, or serum. These tests are easy to perform, involve fewer technical procedures, require small sample volumes that can be obtained through capillary/digital puncture, and have a short processing time, providing rapid results (in 10–35 minutes) without the need for a dedicated laboratory, infrastructure, equipment, and skilled operators. Furthermore, they can be performed at the primary care level, close to the community, thus increasing adherence to treatment. In general, these are immunochromatographic tests (ICT), also known as lateral flow assays (LFAs), that provide a qualitative result [13,14]. Because of these characteristics, rapid tests are of great value in public health, as they can facilitate access to diagnosis, and enable case management in resource-limited and point of care (POC) settings.

RDTs are widely used in the screening of different infections [15]. For example, human immunodeficiency virus (HIV), syphilis, and hepatitis B, along with Chagas disease, are included in the Framework for Elimination of Mother-to-Child Transmission Initiative (EMTCT Plus) [16]. However, while systematic screening with RDTs is used for the other three diseases in the EMTCT Plus framework, this is not yet the case for Chagas disease. PAHO/WHO issued a strong recommendation for using ICT tests in population-based studies to assess the prevalence of Chagas disease [2]. Furthermore, rapid tests are an important part of control programs for other tropical diseases such as malaria, and other neglected diseases including dengue and leishmaniasis have rapid tests available and incorporated into PAHO diagnostic recommendations.

In the last two decades, several RDTs for Chagas disease have been developed, and several studies have assessed their performance in different populations and sample types, with variable results. Such variability could be partially explained by genetic variation in *T*. *cruzi*, leading to differences in infection pathogenicity and transmission. Seven genetic lineages of the pathogen (discrete typing units, DTUs) have been identified to date, with different geographical distributions [17]. TcI is most common in human infections in Colombia [18].

Therefore, the INS, DNDi, and FIND (Foundation for Innovative New Diagnostics) conducted a study to assess the diagnostic performance of 11 rapid tests for Chagas disease in the Colombian population, in order to estimate their operational characteristics under controlled laboratory conditions using the Colombian algorithm for serological diagnosis as a reference standard, and to model potential combinations of RDTs for screening and confirmatory diagnosis of *T*. *cruzi* infection. The tests with the best performance were identified based on their operational characteristics and will be reassessed in further studies performed under field conditions.

## Methodology

The aim of this study was to assess, under controlled laboratory conditions, the diagnostic performance in terms of accuracy, sensitivity, specificity, positive and negative likelihood ratios, false positive (FP) and false negative (FN) rates and true positive (TP) and true negative (TN) rates, of 11 RDTs for the serological diagnosis of *T*. *cruzi* infection. Our hypothesis was that the diagnostic performance of RDTs should not be different when compared with the serological laboratory-based tests used as reference standards in Colombia.

### Study type and design

A retrospective analytical observational study of diagnostic tests was performed to estimate the operational characteristics of 11 commercially available RDTs designed for in vitro detection of anti-*T*. *cruzi* IgG antibodies. The study was developed at the National Reference Laboratory of Parasitology of the INS, which has ISO17025 accreditation by the National Accreditation Body of Colombia (ONAC) for performing selected serological laboratory-based tests. Variables such as sample volume, room temperature and relative humidity, reading time, and storage conditions were strictly controlled according to each manufacturer’s specifications. Moreover, the tests were processed by laboratory personnel using equipment with strict protocols of maintenance and calibration. Each rapid test was performed in compliance with the technical procedures established by the manufacturers. The reference for performance comparison was the current national algorithm for serological diagnosis of chronic infection [19,20].

### Samples

The samples used in this study consisted of human serum previously assessed at the INS with the reference standard. Inclusion and exclusion criteria are as follows. Samples from Colombian patients with suspected chronic *T*. *cruzi* infection collected between January 2019 and March 2021 that had been properly stored (at -70°C) at the INS serum storage facility and for which there was the patient’s informed consent were included. Samples stored in suboptimal conditions or not properly identified, of insufficient volume, in poor technical condition (such as those with fibrin remnants or haemolysis) or with inconclusive results in the reference tests were excluded from the study.

**Fig 1:**
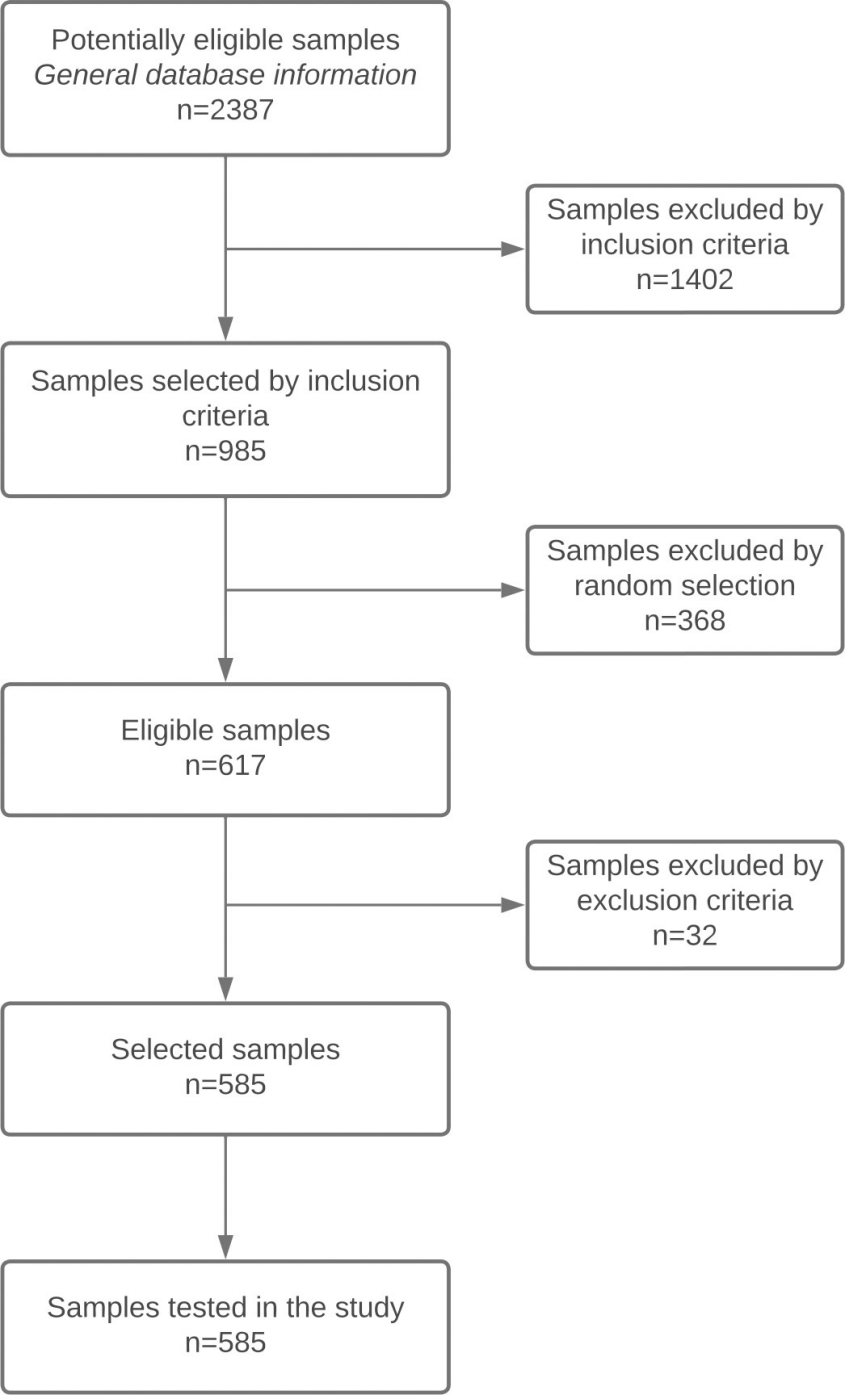
Sample selection flowchart.

The serum samples were collected from both symptomatic and asymptomatic patients, while some of them came from the national quality control programs carried out by the INS in departmental public health laboratories; for this reason, the origin, sex, and age of subjects were not recorded in all cases. Samples were selected using the database of the Chagas Programme of the National Reference Laboratory of Parasitology. Inclusion criteria were applied, and 617 samples were then selected randomly from this group using SPSS. Subsequently, 32 samples were excluded based on exclusion criteria for a final sample size of 585 (Fig 1). Samples with different optical densities and titres were selected, ranging from densities near the cut-off point to maximum densities. Specifically, serum absorbencies ranged from 0.010 to 2.950 as measured by an ELISA reader during the processing of samples according to the national reference standard. Samples representing all ranges were included, though previously indeterminate samples were excluded. To process the rapid tests, we utilized additional supplies which were not included with the test kits, including calibrated pipettes, chronometers, disposable points for pipettes, and protective personal equipment for the operators. All tests were processed according to manufacturer instructions.

### Sample calculation and parameters assessed

Sample size calculation was based on independent calculations for sensitivity and specificity using Tilaki’s formula [21]. For an expected sensitivity of 96% (100–92.5%) and specificity of 98.0% (100–93.0%), a minimum sample size of 501 samples was calculated; however, given the availability of samples and reagents, a total of 585 samples were used; 302 positive (51.6%) and the remaining 283 negative. The parameters assessed for each RDT against the reference standard included accuracy (TP/FN)/(FP/TN) Sensitivity (TP/TP + FN), specificity (TN/TN + FP), false positive (FP/FP+TN) and false negative rates (FN/FN+TP), and positive (sensitivity/1-specificity) and negative (specificity/1-sensitivity) likelihood ratios. Test validity (invalid result rate) was assessed for each RDT, as an analytical performance parameter.

### Reference standard and sample classification

As a reference standard, this study used the current Colombian algorithm for serological diagnosis based on two commercial IgG ELISA assays, based on total (ELISA Chagas III—BiosChile) and recombinant (Elisa–Vircell / Chagatest recombinante 4.0—Wiener Lab) antigens with confirmed sensitivity and specificity >98% [20] for the detection of anti-*T*. *cruzi* antibodies and an "in house" indirect immunofluorescence assay as a tiebreaker in case of disagreement. All techniques underwent secondary validation by the INS. The serological external quality assurance panel, WHO international standard reference panel for anti-*Trypanosoma cruzi* I and II antibodies NIBSC code: 11/216 (NIBSC, UK), was used to corroborate the detection capacity of the 11 RDTs with an international standard.

### Selection of RDTs

The selection of RDTs that was included in the study considered commercial availability in Colombia, current registration with the National Institute for Drug and Food Surveillance (INVIMA), performance in previous publications, and regional production in Latin America of the RDTs. Tests that had not been previously registered with INVIMA were authorized directly by the INS Directorate of Public Health Networks (DRSP). A total of 11 RDTs were evaluated (Table 1).

**Table 1:** Technical characteristics of RDTs included in the study.

RDT	Name	Manufacturer/ country	National Health Registration	Viable sample	Volume WB (uL)	Reading time (min)	Required diluent volume (uL)	Se (%)*	Sp (%)*
ADBIO	Chagas Ad-Bio Combo Rapid Test en Cassette	CTK Biotech/China	Yes	S, P, WB	40-50uL	15 or less	70–100	92.9	100
ART	Chagas Ab Rapid Test	Artron/Canada	Yes	S, P	5uL**	20 or less	100	NA	NA
FIRST	Chagas Rapido First Response	Lemos Lab/Argentina	No data	S, P, WB	10uL	20–30	40	92.2	98.5
HEXA	Hexagon Chagas	Human/France	No	S, P, WB	50uL	10–15	150	97.4	97.2
PLUSRT	Chagas Detect Plus Rapid Test	InBios Inc./USA	No	S, WB	5uL	20	40uL of each one (2)	100	100
SD-AB	SD Chagas Ab Rapid	Standard Diagnostic/Korea	Yes	S, P, WB	100uL	15	No	99.2	100
STATPAK	Chagas Stat-Pak assay	Chembio/USA	Yes	S, P, WB	10uL	15	240	98.5	96.0
TR	TR Chagas	Bio Manguinhos/Brazil	No	S, P, WB	10uL	15–20	3 drops	99.4	98.5
TRYP	Trypanosoma Detect Rapid Test	InBios Inc./USA	No	S, WB	20uL	10	150–200	100	100
WL	WL Check Chagas	Wiener Lab/Argentina	Yes	S, P, WB	40uL	25–35	100	98.6	98.4
XERION	Chagas Ab Xerion Cassette	Xerion/Colombia	Yes	S, P, WB	50uL	15 or less	80	92.9	100

* Reported by the manufacturer.

** Serum or plasma.

RDT = rapid diagnostic test, Se = sensitivity, Sp = specificity, NA = not available, S = serum, P = plasma, WB = whole blood

### Assessment of ease of use

The study assessed four essential elements related to how each test is used. The objective was to assess and report the experience of using each test from the operators’ perspective. Operators (highly skilled laboratory technicians at INS) gave their subjective assessment of the following elements: 1) the appearance of the test device background once the sample was added (if the background was “clear” the test obtained a higher score compared to “dark” as it increases contrast, and thus readability), 2) the intensity at which the control/test bands were colored (if the control/test bands were clear and dark, the test obtained a higher score compared to bands that were light, faint or diffuse), 3) the quality and comprehensiveness of the package insert (instructions for use declared by the manufacturer or data sheet, and 4) ease of observing the result (evaluation of the device background and band intensity combined). To measure the operators’ assessments, a value was assigned to each element in each category The score in each category was determined by consensus between the two operators who processed the tests. A total score between 5 and 12 was calculated by summing the scores from each category, with 12 representing the greatest user-friendliness. Additionally, we checked whether each RDT included a sample dispenser in its commercial packaging.

### Data collection and analysis

The primary data and photographic images were recorded with the TiraSpot mobile app (SpotLab, Spain) for smartphone per operator. The RDT images and associated information were anonymized and securely stored in the TeleSpot cloud platform (Spotlab, Spain) from which a single database combining the metadata and the photographs was created [22]. Furthermore, physical records endorsed by the INS quality assurance system were used. All tests were validated by two blinded operators. In case of disagreement between the first two operators, a third operator decided the case. Discordant results between the rapid tests and the reference standard were analyzed individually; samples presenting discordant results in more than five RDTs were reprocessed using the reference tests to validate their final classification. Univariate and bivariate statistical analyses were performed for each RDT using SPSS v18 (IBM, Armonk, NY, USA). The point estimate (%) and 95% confidence intervals for accuracy, sensitivity, specificity, false positive rate, false negative rate, and positive and negative likelihood ratios were calculated using Epidat 3.0 (Dirección Xeral de Saúde Pública, Galicia, Spain), Microsoft Excel v16.61.1 (Microsoft Corporation 2022) and R Software (www.r-project.org) (Boston, MA, USA).

### Ethical considerations

A total of 555 patients provided their signed informed consent at the time of sample collection. The remaining 30 samples included in the study came from blood donors at Colombian blood banks without prior consent, as they were samples that entered the INS for quality control and, according to Colombian regulations, could be used anonymously in studies related to the mission of the National Reference Laboratory. Data recording used a coding system which could only be accessed by the researchers and/or authorized personnel. The data recorders were INS professionals subject to a confidentiality agreement in accordance with article 34 of Law 23 (1981). The research carried out in this study was considered as minimal risk research, in accordance with Resolution 8430 (October 4th, 1993) of the Ministry of Health.

## Results

The patient’s age was only available in 28.3% (166) of the samples; the mean age was 32.5 years (SD = 22.4), ranging from 1 to 81 years. In 44.1% (258) of the samples, the patient’s gender was recorded; 55.8% (144) were female and 44.2% (114) were male. Samples from most (19/32) Colombian departments were included, with highly endemic areas such as Arauca, Santander, Boyacá, and Casanare, as well as Bogotá—a non-endemic area without active vector transmission (Table 2).

**Table 2:** Department of origin of included samples.

Department of origin	n	%*
Arauca	147	25.1
Santander	71	12.1
Boyacá	60	10.3
Bogotá**	37	6.3
Caquetá	28	4.8
Casanare	37	6.3
Sucre	28	4.8
Cundinamarca	21	3.6
Bolívar	13	2.2
Norte de Santander	9	1.5
Atlántico	8	1.4
Meta	8	1.4
Tolima	6	1.0
Putumayo	5	0.9
Cesar	3	0.5
Chocó	3	0.5
Antioquia	2	0.3
La Guajira	2	0.3
Córdoba	1	0.2
Valle	1	0.2
Not available	95	16.2
**Total**	**585**	**100**

*Proportion of samples from this department.

** The only area without active vector transmission.

All 585 samples were analyzed using the 11 RDTs, except for the TR, which was only used to analyze 551 samples because of a lack of reagents due to problems related to the assay datasheet. The samples represented a broad range of optical densities on the national reference standard (S1 Fig). Of the 585 total samples used in the study of which 302 were originally classified as positive and 285 negative by the national reference standard, 61.4% (359) presented concordant results across all the RDTs evaluated. Of the samples with concordant results, 45.4% (163) had previously been characterized as positive and 54.6% (196) as negative. In total, 38.6% (226) of the samples had at least one discordant RDT result; 61.5% (139) were in samples previously characterized as positive and 38.5% (87) as negative, i.e., most of the discordant results were false negatives. According to a chi squared test, the different rates of discordance between samples which were previously characterized as positive or negative was significant (p≤0.05).

Of the 6,401 tests analyzed, 7.4% (471) disagreed with the reference standard, of which 75.8% were false negatives and 24.2% (114) were false positives. Samples with discordant results between rapid tests and the national reference standard tended to present values which were nearer to the cut-off point of the total antigens ELISA used in the latter. We tested the hypothesis that discordance between the rapid tests and the national reference standard in a given sample would be driven by the amount of antibody titers. Samples which were discordant between the reference standard and rapid tests tended to have lower values, but this was not statistically significant. Therefore, discordance between the rapid tests and the national reference standard did not appear to be correlated with the amount of antibody titers in the samples.

### Operational characteristics of the RDTs

**Fig 2:**
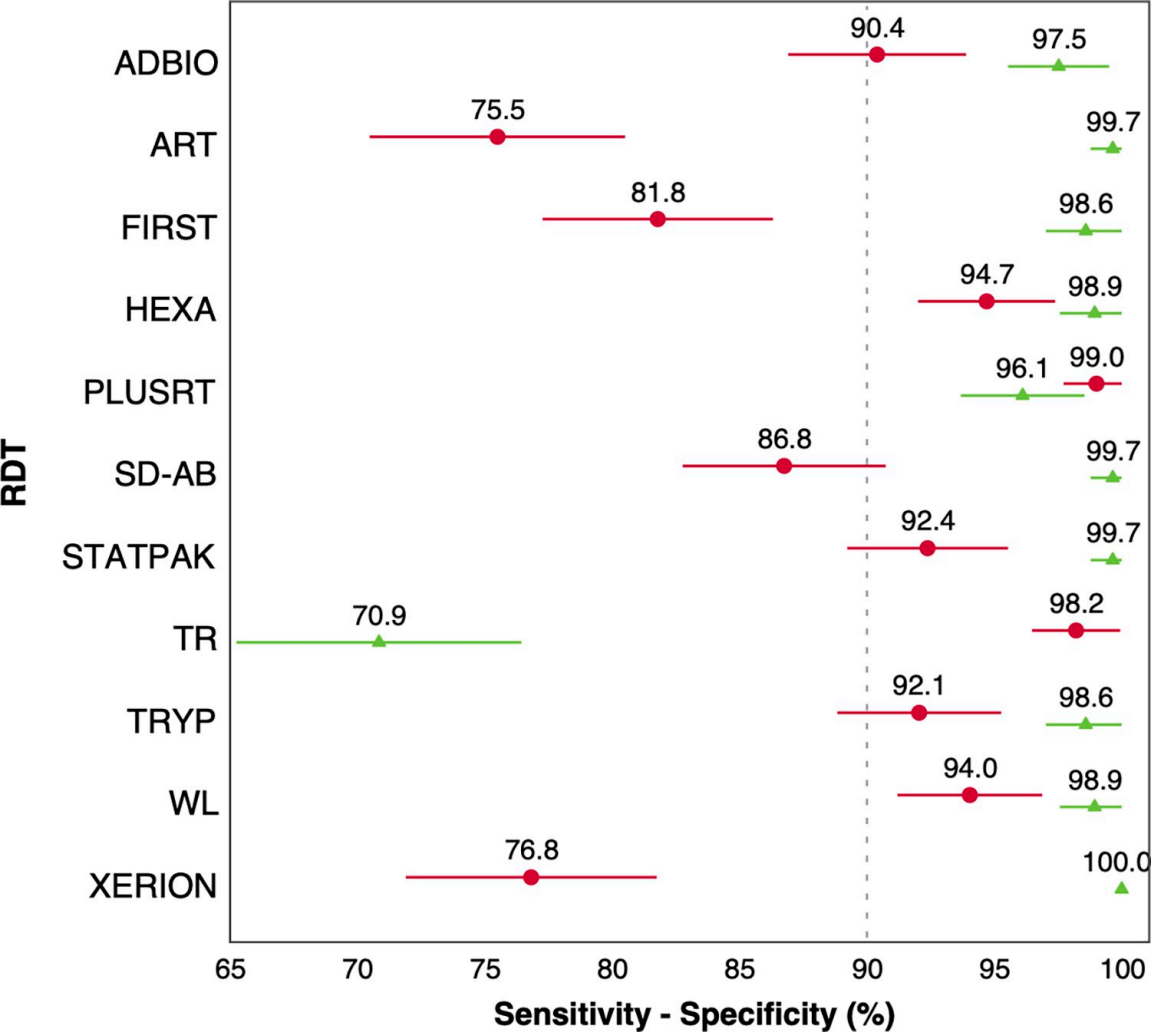
Operational characteristics of 11 RDTs. Δ Sensitivity (%). ○ Specificity (%). Horizontal bars indicate the 95% confidence interval.

Table 3 shows the operational characteristics of the 11 RDTs assessed in this study. Using the current diagnostic algorithm for chronic infection in Colombia as a reference standard, the sensitivity of the assessed RDTs ranged from 75.5% to 99.0% (95% CI 70.5–100), while specificity ranged from 70.9% to 100% (95% CI 65.3–100). Most tests (7/11, 63.6%) had sensitivity above 90%, and almost all (10/11, 90.9%) had specificity above 90%. Two of the 11 tests (18.2%) had sensitivity between 98 and 100% (95%CI 96.5–100), while 8 (72.7%) had specificity within that range (95% CI 97.0–100) (Table 3 and Fig 2).

**Table 3:** Operational characteristics of the assessed RDTs.

RDT	Accuracy % (95%IC)	Sensitivity % (95%IC)	Specificity % (95%IC)	FPR (%)	FNR (%)	LR+ (95% IC)	LR- (95% IC)	Proportion of invalid n (%)
ADBIO	93.85	90.4	97.53	2.47	9.6	36.55	0.1	2 (0.3)
	*(91*.*8-95*.*9)*	*(86*.*9-93*,*9)*	*(95*.*5-99*.*5)*			*(17*.*6-76*.*0)*	*(0*.*07-0*.*14)*	
ART	87.18	75.5	99.65	0.35	24.5	213.66	0.25	0 (0)
	*(84*.*4–89*.*9)*	*(70*.*5–80*.*5)*	*(98*.*8–100)*			*(30*.*2–1513*.*1)*	*(0*.*2–0*.*3)*	
FIRST	89.91	81.79	98.59	1.41	18.21	57.87	0.18	3 (0.5)
	*(87*.*4–92*.*4)*	*(77*.*3–86*.*3)*	*(97*.*0–100)*			*(21*.*8–153*.*3)*	*(0*.*15–0*.*23)*	
HEXA	96.75	94.7	98.94	1.06	5.3	89.34	0.05	1 (0.2)
	*(95*.*2–98*.*3)*	*(92*.*0–97*.*4)*	*(97*.*6–100)*			*(29*.*0–275*.*4)*	*(0*.*03–0*.*09)*	
PLUSRT	97.61	99.01	96.11	3.89	0.99	25.47	0.01	0 (0)
	*(96*.*3–98*.*9)*	*(97*.*7–100)*	*(93*.*7–98*.*5)*			*(14*.*3–45*.*5)*	*(0*.*00–0*.*03)*	
SD-AB	92.99	86.75	99.65	0.35	13.25	245.52	0.13	4 (0.7)
	*(90*.*8–95*.*2)*	*(82*.*8–90*.*7)*	*(98*.*8–100)*			*(34*.*7–1737*.*8)*	*(0*.*10–0*.*18)*	
STATPAK	95.9	92.38	99.65	0.35	7.62	261.45	0.08	6 (1.0)
	*(94*.*2–97*.*6)*	*(89*.*2–95*.*5)*	*(98*.*8–100)*			*(37*.*0–1850*.*1)*	*(0*.*05–0*.*11)*	
TR	84.75	98.21	70.85	29.15	1.79	3.37	0.03	0 (0.0)
	*(81*.*7–87*.*9)*	*(96*.*5–99*.*9)*	*(65*.*3–76*.*4)*			*(2*.*8–4*.*1)*	*(0*.*01–0*.*06)*	
TRYP	95.21	92.05	98.59	1.41	7.95	65.13	0.08	0 (0.0)
	*(93*.*4–97*.*0)*	*(88*.*8–95*.*3)*	*(97*.*0–100)*			*(24*.*6–172*.*4)*	*(0*.*05–0*.*12)*	
WL	96.41	94.04	98.94	1.06	5.96	88.71	0.06	1 (0.2)
	*(94*.*8–98*.*0)*	*(91*.*2–96*.*9)*	*(97*.*6–100)*			*(28*.*8–273*.*5)*	*(0*.*04–0*.*09)*	
XERION	88.03	76.82	100	0	23.18	-	0.23	0 (0.0)
	*(85*.*3–90*.*8)*	*(71*.*9–81*.*8)*	*(99*.*8–100)*				*(0*.*19–0*.*28)*	

FPR, false positive rate; FNR, false negative rate; LR+, positive likelihood ratio; LR-, negative likelihood ratio; numbers indicate the point estimate (%) with 95% confidence interval

### Assessment of user-friendliness

User-friendliness was assessed using a score that represented the operator appraisal of the four elements mentioned above and took into account the volume of blood and the design of the sample dispenser (Table 4). The WL Check Chagas test (Wiener Lab, Argentina) had the highest score, thus being considered the most user-friendly. Other findings included the following: the Chagas Rapid First Response test (Lemos Lab, Argentina) can produce a red spot in the Test band (T-band) in some negative tests, which may confuse inexperienced or poorly trained operators; Trypanosoma Detect Rapid test (Inbios Inc., USA) requires specific additional supplies to perform the test; and for Chagas Ab Xerion Cassette test (Xerion, Colombia) not every red line in the T-band indicates a positive sample, as the T-line must be at least as intense as the C-band, which may also generate some uncertainty for inexperienced operators. In five tests a dark/stained background was observed in some cases, which may interfere with the test’s readability. Band intensity varied across RDTs, and it was often weak in four of the eleven RDTs. The quality of the package insert (manufacturer instructions for use) was considered “fair” in one test, while in two tests it was “difficult to read”. A sample dispenser was included in seven RDTs. The evaluation of these criteria represents the subjective opinions of the three highly skilled laboratory technicians who assessed the tests; individual user experience may vary from this.

**Table 4:** Assessment of ease of use.

RDT	Appearance of the background in the device after testing^a^	T/C band intensity^b^	Quality of package insert^c^	Ease of reading^d^	Sample dispenser included in the kit^e^	Score
ADBIO	1	2	2	3	2	10
ART	2	1	2	2	1	8
FIRST	1	1	3	1	2	8
HEXA	1	2	2	2	2	9
PLUSRT	2	2	2	2	1	9
SD-AB	2	1	3	2	1	9
STATPAK	1	2	2	3	2	10
TR	1	2	3	2	2	10
TRYP	2	2	2	2	1	9
WL	2	2	3	3	2	12
XERION	2	1	1	1	2	7

^a^ 2 = clear, 1 = dark

^b^ 2 = frequently intense, 1 = frequently weak

^c^ 3 = very good, 2 = good, 1 = fair

^d^ 3 = effortless, 2 = difficult, 1 = very difficult

^e^ 2 = yes, 1 = no

score = summation of each variable

### Test validity

The data sheet provided by the manufacturer of each RDT describes the conditions that the test must meet for the result to be considered valid. These conditions were taken strictly into account, and between 0.0% and 1.0% of test results were invalid. (Table 3). Five of the 11 RDTs had no invalid results, two tests had 0.2% invalid results, three had between 0.3% and 0.7%, and one test had 1% invalid results.

## Discussion

The present study shows that in terms of diagnostic performance, 6/11 rapid tests had sensitivity and specificity above 90%, while two had sensitivity above 98%. Eight of the 11 tests also had specificity above 98%. The results of our study will be used to select at least two of the rapid tests for a future field study–depending not only on the characteristics described here but also on their availability in the market and feasibility of implementation in the healthcare system. The results of this study showed that discrepancies between RDTs and reference tests occurred more frequently in samples previously characterized as positive, indicating that most of these RDTs may fail in detecting the analyte (anti-*T*. *cruzi* antibodies) present in the samples, a phenomenon that some manufacturers indicate in their datasheets and attribute to the limit of detection (LOD) of the test, which is not specified by the manufacturers. This phenomenon is inherent to the analytical method and technology type. Further research is required to clarify this question; one potential direction could be comparing the lowest concentration of the analyte in a sample that can be consistently detected with a stated probability (LOD) per test.

The other variables used in the panel, such as age, sex, and sample origin, were not statistically related with the level of agreement between the techniques and were apparently not associated with the success or failure of the RDTs. However, data on sex and age was not available for the majority of the samples.

The optical density (OD) of each sample is given by the absorbance in the reference tests (ELISA methods with total or recombinant antigens). Our study did not find a statistically significant association between variations in the OD of the reference test results and performance of the RDTs. It is generally believed that samples with analyte (anti-*T*.*cruzi* antibody) levels near the cut-off point are more likely to produce discordant test results, due to the limited amount of analyte in the sample; however, in the present study, this was not observed. No differences were found between discordant and concordant results of the RDTs with the variation of analyte levels.

The processing of rapid diagnostic tests is technically undemanding; however, such tests must comply with standard biosafety requirements and the manufacturer’s instructions must be easy to follow. Qualitative assessments of the user-friendliness of RDTs can give insight into real world experience and provide useful information for evaluating incorporation of RDTs into a potential diagnostic algorithm for Chagas disease.

Our study contributes to a growing body of literature supporting the feasibility of using rapid tests in the diagnosis of Chagas disease. In a collaborative study between the WHO, Doctors Without Borders, and national reference laboratories, Sanchez-Camargo and colleagues (2014) also evaluated 11 rapid tests using 474 samples obtained from several endemic and non-endemic countries [14]. Similar to our study, Sanchez-Camargo et al. [14] used previous classification by national reference laboratories following WHO recommendations (two concordant results on two different serological assays). They found that most (6/11) tests had a sensitivity greater than 90%, while 9/11 had a specificity of between 90 and 100%. There were no significant geographical variations in test performance between countries. Our study included four of the rapid tests evaluated in this study: Check Chagas (Wiener Labs), Chagas Stat-Pak Assay (Chembio), Trypanosoma Detect Rapid Test (InBios) and SD Chagas AB Rapid (Standard Diagnostics). Results were comparable; as in our study, all four exhibited specificity >90%. However, we are unaware of any updates or changes in the technical configuration of the assays that might have been made in the ten-year gap between the studies.

A systematic review performed by Angheben and colleagues (2019) included 10 studies that assessed six different RDTs. The overall sensitivity was 96.6% (95% CI 91.3–98.7%) and the overall specificity was 99.3% (95% CI 98.4–99.7%), with the highest values found in endemic areas [23]. The authors supported the inclusion of RDTs in the diagnostic process, potentially combined with a laboratory test for confirmation. Other studies have also compared the use of commercially available rapid tests to laboratory-based serological techniques. In Argentina, an evaluation of two rapid tests approved for use in the country was performed on 607 samples, using three serological tests included in national guidelines as a reference standard [24]. In that study, SD BIOLINE Chagas Ab Rapid test (Abbott-Standard Diagnostic, USA) achieved sensitivity of 97.2% and specificity of 91.7%, while Wiener Labs Check Chagas achieved 93.4% and 99.1%, respectively. The latter test had values similar to those in our study. In another assessment conducted at point-of-care settings in Boyacá, a Chagas disease endemic department in Colombia, the authors evaluated two RDTs for case definition, the tests included were the Chagas Stat-Pak assay and Chagas Detect Plus Rapid Test, using two ELISA tests and an IFA test as reference standards. Both RDTs had sensitivity and specificity greater than 99% [25]. Agreement between the two RDTs was 99.5%. In addition, in a study with 106 serum samples in Argentina, sensitivity was greater than 97% and specificity approached 100% when these two rapid tests were assessed, with disagreement of 6.6% between the two tests [26].

Another important factor to be considered is the potential for geographic variation in the performance of diagnostic tools. Truyens and colleagues (2021) evaluated the performance of rapid and serological tests in the diagnosis of *T*. *cruzi* in 481 samples collected from women in Honduras, Mexico, and Argentina, confirming the reactivity of the samples by PCR. The performance of all tests varied significantly among countries, with the worst performance in samples from Mexico. However, when two rapid tests were used in combination, performance was comparable to ELISA techniques. The authors concluded that the differences in test performance between countries were not due to differences in parasitemia, but rather to differences in antibody levels against ELISA antigens [27].

Other studies have evaluated rapid test performance in dogs. Rodrigues et al. assessed the Bio-Manguinhos Lateral Flow Immunochromatographic Rapid Test in 281 serum samples from domestic dogs and 9 from wild canids in Brazil [28]. The authors found a significant correlation between the intensity of bands and the antibody titers from prior serological analyses. Cross reactions were observed in samples infected by *Crithidia mellificae*, *Anaplasma* sp. and *Erlichia* sp. In another study examining the incidence of *T*. *cruzi* infection in dog kennels in Texas, both Chagas Stat-pak and InBios Chagas Detect Plus were used, with the former showing high agreement with an immunofluorescence assay (kappa = 0.84) [29]. Both tests, while designed for humans, have been employed in research studies of *T*. *cruzi* infection in dogs and cats [29,30].

Rapid tests have been effectively employed to provide immediate point of care diagnosis for other infectious diseases, notably HIV [15,31]. RDT duos–using two RDTs simultaneously—are a promising option which could provide diagnostic confirmation of chronic infection at the point of care. This could be particularly valuable for vulnerable populations that face challenges in accessing healthcare. Some studies have already assessed the use of paired RDTs in Chagas disease endemic countries, with results comparable to those obtained using laboratory-based algorithms [32,33].

Our study has some limitations. As there is no regional gold standard for diagnosing *T*. *cruzi* infection, we used the Colombian diagnostic algorithm as a reference standard to determine true positives and negatives. The same algorithm had been previously evaluated using an in-house ELISA and IFA, both based on Colombian *T*. *cruzi* strains, an indirect haemagglutinin assay, and a trypomastigote excreted-secreted antigens assay (TESA), with a reported sensitivity above 98% and specificity approaching 100% [20]. Furthermore, the biological matrix of samples used in our study was serum, but results may vary when whole blood is used. Another limitation is that we did not incorporate polymerase chain reaction (PCR) or other parasitological methods, which have low sensitivity for detecting chronic *T. cruzi* infection, or parasitologically negative controls. We also did not have samples available with confirmed infection by *Leishmania* spp. which met inclusion criteria in order to assess the impact of cross-reaction, nor did we analyze samples which were indeterminate according to the national reference standard (in which case an immunofluorescence assay is used as a tiebreaker). This was a laboratory-based study where environmental conditions such as storage temperature, processing temperature, relative humidity, and time were strictly controlled, and the measurements were made by highly qualified personnel using calibrated equipment; however, results may vary when the tests are performed in primary care centers and/or in the community. Due to geographical variation in diagnostic performance and differences in the types of circulating DTUs across countries, our results may not be applicable to other areas.

In conclusion, our study demonstrates that several rapid tests can have a performance comparable to the Colombian diagnostic algorithm, which is based on ELISA serological tests. The failure to systematically diagnose *T*. *cruzi* infection is one of the main causes of persistent neglect towards this global public health problem more than 100 years after its discovery, the epidemiological silence around the disease persists, and most patients remain undiagnosed and untreated. Because most patients are unaware they are infected, active screening of people at risk through primary healthcare centers is an urgent need. Rapid tests could simplify the diagnostic process for patients and healthcare providers, potentially providing an immediate result at the point of care, and allowing immediate evaluation to start a treatment regimen, avoiding delays in patient management and loss to follow-up. Ultimately, comprehensive control of vector, congenital, and other routes of transmission will be key to achieving the World Health Organization objective of eliminating Chagas disease as a public health problem by 2030 [34].

In order to implement the systematic use of rapid tests in the field or at point of care settings, further studies must be conducted to evaluate the performance of RDTs using whole blood and in field conditions. Furthermore, it is important to determine whether combinations of rapid tests used in pairs can be useful for early confirmation of the diagnosis in populations at risk of *T*. *cruzi* infection. This could improve access to early diagnosis and treatment for this underdiagnosed and neglected disease, especially among marginalized and vulnerable communities.

## Supporting information

S1 Fig Distribution of optical densities of the 585 samples included in the study according to their reference value. (PDF)

S2 Fig Distribution of the optical densities of the 585 samples included in the study according to RDT result. (PDF)
