# Supplementary figures and images for: Laboratory evaluation of eleven rapid diagnostic tests for serological diagnosis of Chagas disease in Colombia
Source: PLoS Negl Trop Dis. 2023 Aug 22;17(8):e0011547. doi: 10.1371/journal.pntd.0011547 (PMC10473487; doi:10.1371/journal.pntd.0011547)

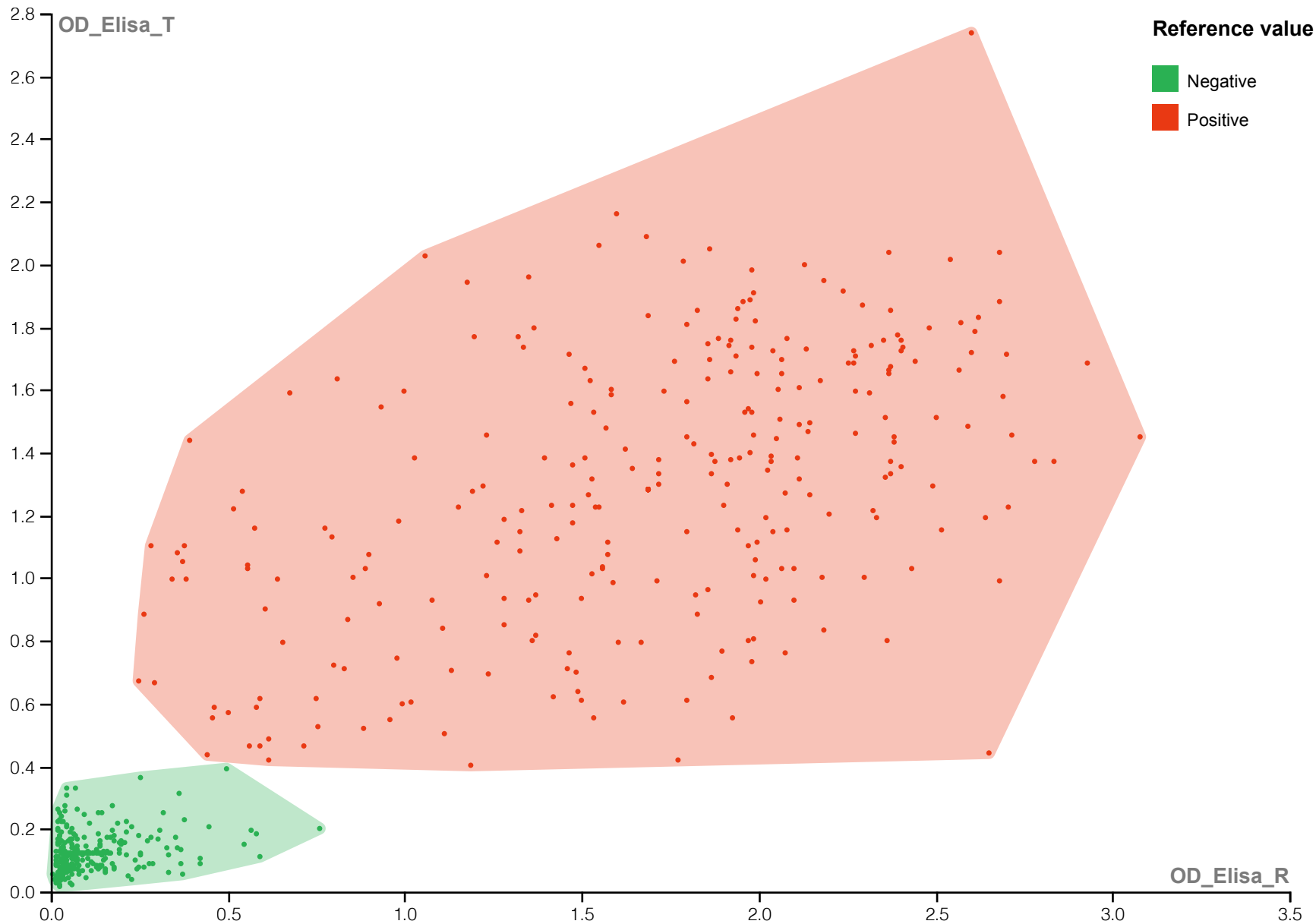

Supplement: S1 Fig — (PDF) [file pntd.0011547.s001.pdf]

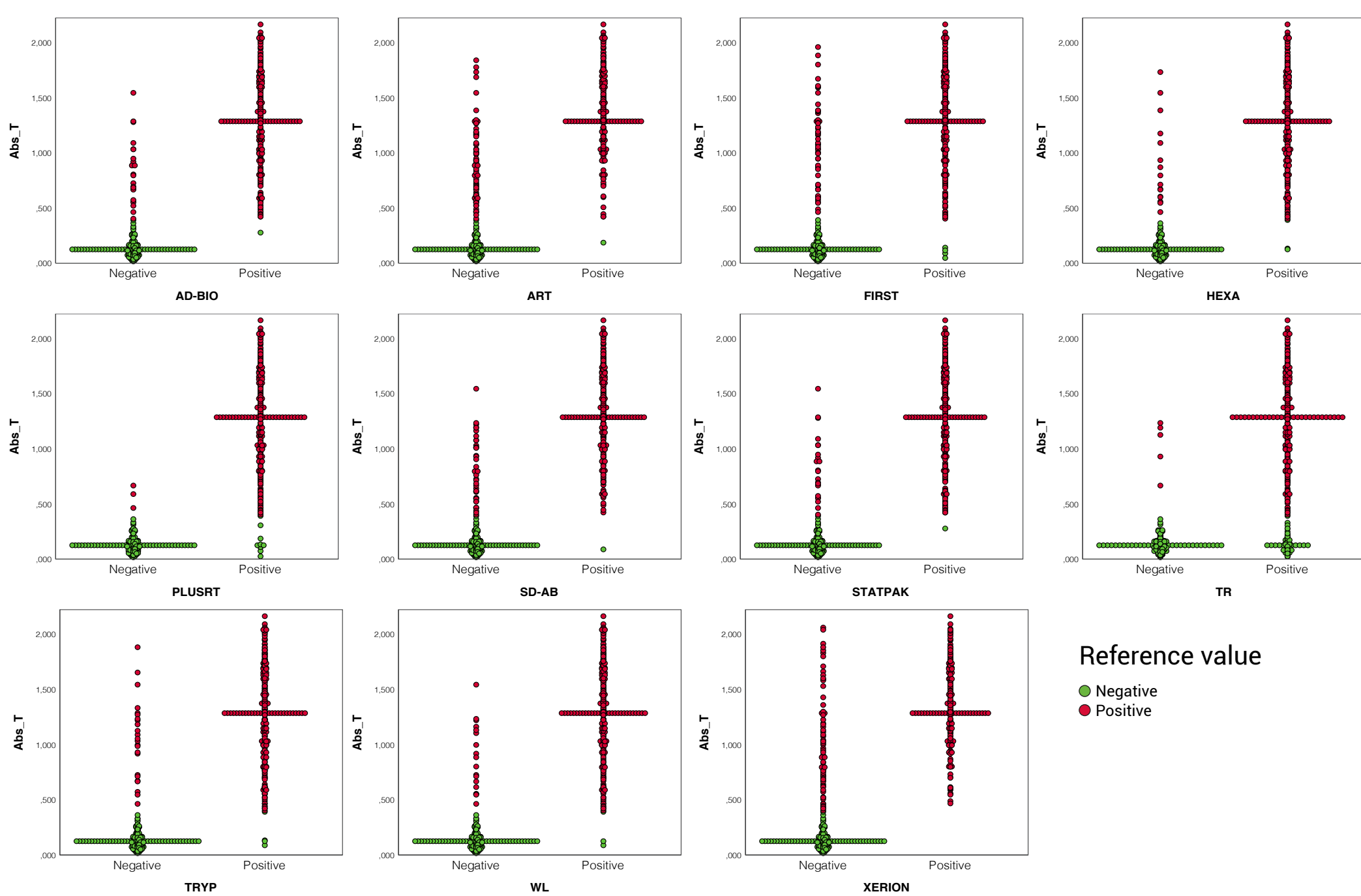

Supplement: S2 Fig — (PDF) [file pntd.0011547.s002.pdf]
